# Validation of the Component Model for Prediction of Moisture Sorption Isotherms of Two Herbs and other Products

**DOI:** 10.3390/foods8060191

**Published:** 2019-06-01

**Authors:** Chiachung Chen

**Affiliations:** Department of Bio-industrial Mechatronics Engineering, National Chung Hsing University, 250 Kuokuang Road, Taichung 4022, Taiwan; ccchen@dragon.nchu.edu.tw; Tel.: +886-4-22857562; Fax: +886-4-22857135

**Keywords:** sorption isotherms, component model, chrysanthemum morifolium, *Anoectochilus formosanus* Hayata

## Abstract

Sorption isotherm is an essential property for the processing of biological materials. In this study, a component model for the prediction of the sorption isotherm was evaluated. In order to validate this component model, the moisture sorption isotherms for *Chrysanthemum morifolium* flowers and the orchid *Anoectochilus formosanus* Hayata were determined. The sorption isotherm was measured by using the equilibrium relative humidity technique for five temperatures. Seven sorption isotherm models were selected with four quantitative criteria and residual plots to evaluate fitting ability and prediction performance for these products. The results indicated that the sorption temperature did not significantly affect the adsorption isotherm. The Caurie and Henderson equations could be used for *C. morifolium* flowers and *A. formosanus* Hayata. The isotherm data of raw bamboo, elecampe and three varieties of corn kernels from the literature were adopted to validate the component model. Comparing with the predicted values of component values and actual isotherm moisture, the component model has good predictive ability at the a_w_ range smaller than 0.7. Considering the practical application, the a_w_ range below 0.7 is the main range for the processing of agricultural products, and the predictive values of this component model could be helpful for food engineering and for the food industry.

## 1. Introduction

Living plants need to be processed after harvesting. Processing operations include drying, packaging, handling, and storing. The physical, chemical and biological conditions such as microbial growth of plant materials are affected by moisture content, temperature and relative humidity (RH) of the ambient environment and treatment methods [1,2]. 

At fixed temperatures and pressures, the relationship between the relative humidity (RH) of ambient air and moisture content is called sorption isotherm. The definition of the water activity (a_w_) is the ratio between the partial vapor pressure of water in the biological materials and the partial vapor pressure of pure water at the same temperature. The equilibrium relative humidity is called water activity in food science field. The sorption isotherm dictates the corresponding water content at the same temperature for each humidity. Sorption isotherm properties include desorption and adsorption according to the adsorption or dehydration of moisture content. The sorption isotherm is essential information for the predicting drying and storage of materials. The design of packaging materials and the method are also related to these properties.

There are several methods to determine the sorption isotherms of biological materials [1]. For the gravimetric method, the RH is maintained with different saturated salt solutions or sulphuric acid dilutions at different concentrations. The samples are enclosed in this RH environment at content temperature until its weight is balanced with ambient RH [3,4,5]. This equilibrium moisture content (EMC) method is simple, inexpensive and can be performed in most laboratories. However, the equilibrium time is longer, especially at higher RH. These samples could be contaminated if the ambient RH value is higher than 75 %. The other method is called the hygrometric or equilibrium relative humidity method. The samples at a given moisture content and fixed temperature are placed in an enclosed container. The RH in the container is measured as the humidity environment reaches the equilibrate state [6,7,8]. The key points for this technique are the uniform moisture distribution of samples and the accuracy of the hygrometer.

Crapiste and Rotstein [9] predicted the sorption isotherms of potatoes based on the magnitude and sorption properties of individual constituents. By equating the chemical potential of water in each component and that of water in the surrounding air, equations were derived to calculate the moisture content occupied by each component. In this study, their model was modified to calculate the sorption isotherms of herbs.

Empirical models are useful to quantify the relationship between equilibrium moisture content and equilibrium relative humidity of biological materials. Basu et al. [1] and Al-Muhtaseb et al. [10] provided a detail review of widely used sorption models. 

Literature on moisture sorption isotherm for agricultural and food products is abundant. Bonner and Kenney [11] reported the moisture sorption characteristics of energy sorghum, and Oyelade et al. [5] investigated maize flour. The EMC/a_w_ properties of various plants with medical functions or industrial crops were reported. Argyropoulos et al. [12] introduced the sorption isotherms of leaves and stems of lemon balm (*Melissa officinalis.* L), Bahloul et al. [13] examined Tunisian olive leaves (*Oleu europaea* L.), Choudbury et al. [14] investigated raw bamboo (*Dendrocalamus longispathus*) shoots and Ait Mohamed et al. [3] determined sorption properties of *Gelidium sesquipedale*. The EMC/a_w_ data of conidia of *Beauveria bassiana* (Balsam) Vuillemin was studied by Hong et al. [15].

The flower of *C. morifolium*, one of varieties for making chrysanthemum flower tea, is popularly used as a medicine and is a healthy beverage. The anti-oxidation and anti-ischemia-reperfusion injury action have been shown proved with animal tests and clinic studies [16]. *A. formosanus* Hayata is a folk medicine in Taiwan. According to studies by Shih et al. [17], this herb has anti-inflammation and liver protection effects and could be used to treat hypertension, diabetes mellitus and tuberculosis. 

The objectives of this study were to (1) determine the moisture adsorption behavior for three biological products at five temperature by using the equilibrium relative humidity technique, and, (2) evaluate the fitting ability of six sorption isotherm models to describe the experimental data, (3) evaluate the predictive ability of the component model for two herbs and other agricultural products.

## 2. The Component Model

### 2.1. Development of the Component Model

There are six components are considered: protein, starch, fiber, oil, sugar, and ash. Sugars and ash were combined as the vacuole component. The effect of oil component on the sorption isotherms is omitted due to its insignificance.

The total moisture content is:

H_t_ = H_v_ + X_f_ × X_f_ + X_s_ × H_s_ + X_p_ × H_p_ (1)

where H_i_ = the water content (g of water)

X_i_ = the weight content (decimal)

t, v, f, s and p denote the total materials, vacuole, fiber, starch, and protein.

### 2.2. The Isotherm Equation of Each Component

#### 2.2.1. The Vacuolar Component

The a_w_ (water activity) values of the vacuolar component was calculated by the Ross equation [18],

a_w v_ = a_w g_ × a_w s_ × a_w a_ (2)

where a_w g_ = the a_w_ of glucose

a_w s_ = the a_w_ of sucrose

a_w a_ = the a_w_ of ashes

From the study of Crapiste and Rotstein [9],

a_w g_ = X_g_ × 10^(−0.858*(1 − X_g_)^2.0 (3)

a_w s_ = X_s_ × 10^(−2.772*(1 − X_s_))^2.0 (4)

a_w a_ = X_a_ × 10^(−0.716*(1 − X_a_)^2.0 (5)

where X_g_, X_s_ and X_a_ are the water mole fractions of glucose, sucrose, and ashes.

The water mole fraction can be calculated by:

(6) $$X_{wi}=\frac{X_{v}}{X_{v}+W_{i}\cdot A_{i}}$$

A_i_ = M_w_/M_i_ (7)

where M_w_ = The molecular mass (g/mol) of water

M_i_ = The molecular mass (g/mol) of component

W_i_ = The weight fraction of component

Therefore, X_g_, X_s_ and X_a_ are calculated as follows:

(8) $$X_{g}=\frac{X_{v}}{X_{v}+0.08326\times W_{glucose}}$$

(9) $$X_{s}=\frac{X_{v}}{Xs+0.08764\times W_{sucrose}}$$

(10) $$X_{a}=\frac{X_{v}}{X_{v}+0.16981\times W_{ashes}}$$

a_w_ and X_v_ can then be evaluated with Equations (2)–(10).

#### 2.2.2. Fiber Component

The Kelsey correction equation [19] was used to express the relationship in the water-cellulose-moist air system.

(11) $$a_{w\;\mathrm{fber}}=\left(\frac{X_{f}-y}{X_{f}}\right)\times Exp\left(\frac{0.16137}{X_{f}+0.43684}\right)$$

(12) $$y=(\frac{0.076+X_{f}-\left[({\left(0.076+X_{f}\right)}^{2}-0.28546\times x_{f}\right){]}^{0.05}}{1.878}$$

The relationship for X_f_ and a_w fber_ was computed by Equations (11) and (12).

#### 2.2.3. Starch Component

Original isotherm data for starch have been presented by Chung and Pfost [20]. The Henderson equation was used to describe the relation of X_s_ and a_w_.

X_s_ = 18.19182 × (−ln(1 − a_w_)^0.41181 (13)

#### 2.2.4. Protein Component

Two sets of protein isotherm data were calculated and proposed [21].

X_p_ = 0.090614 × (−1n(a_w_)^−0.62) (14)

For a given a_w_ value, the moisture content of each component can be obtained. The relationship between a_w_ and moisture content of different herbs could be calculated.

## 3. Materials and Methods

### 3.1. Materials

The biological materials, used for this study was purchased at a local herb market, in Taichung, Taiwan. The initial moistures of *C. Morifolium* flower and *A. formosanus* Hayata were 2.05 % and 1.98 % (on dry basis), respectively.

The desired determination moisture content ranged from 2 % to 20 %, the moisture content for packaging, storing, handling and processing. The samples were rewetted by adding predetermining amount of the water to obtain the desire moisture content. The preparation followed the procedure of Shen and Chen [22]. Samples were mixed with water and stored in plastic containers. After mixing, samples were stored at 5 °C for two weeks to ensure uniform distribution of moisture content. Because of the lower storage temperature, no microbial growth was found during the two weeks’ storage.

### 3.2. Temperature and RH Sensors

The temperature and RH probes of the Shinyei THT-V2-112-73-A2 transmitter (Shinyei Technology, Kobe, Japan) was used. The specifications of this sensor are in Table 1.

### 3.3. Calibration of Sensors 

The temperature and RH transmitter was calibrated. The temperature sensing element was calibrated by the TC-2000 temperature calibrator (Instutek AS, Scan-Sense AS, Bekkeveien 163, N-3173 Vear, Norway) and the humidity sensing element was calibrated by several saturated salt solutions. The detail method included the saturated salts that were used, solution volume to air volume ratio and the stable of the temperature were according to the requirement of Organization Internationale De Metrologie Legale (OMIL) [23].

### 3.4. The Equilibrium Relative Humidity Method

**Figure 1:**
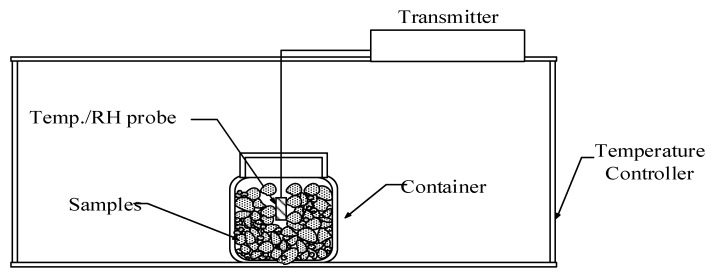
Sketch of experimental set-up.

The moisture sorption isotherms of *C. Morifolium C.* flower, and *A. formosanus* Hayata at five temperatures (i.e., 5 °C, 15 °C, 25 °C, 35 °C, and 45 °C) was determined by the equilibrium relative humidity method. Samples of known moisture content were placed in a 350 mL container. RH/temperature probes were inserted into the containers and surrounded with samples. These sensors and containers were placed in a temperature-controlled chamber. When the RH and temperature within the sample containers reached the equilibrate state, RH and temperature were recorded. The samples were taken out and the moisture content was determined again. Then new samples and containers were placed into a temperature-controlled chamber for the next temperature level. The reading of RH and temperature of these probes was transformed into actual values with pre-established equations to ensure measured accuracy. The set-up for the measurement is presented in Figure 1. This technique has been used to determinate sorption isotherm for peanuts [6], sweet potato slices [24], pea seeds [25], Oolong tea [7] and autoclaved aerated concrete [8].

The moisture content of the samples was determined by using a drying oven at 105 °C for 24 h.

### 3.5. Moisture Sorption Isotherm Models

Seven sorption isotherm equations were used to evaluate the fitting ability and prediction performance of sorption isotherms of *C. Morifolium* flower and *A. formosanus* Hayata at five temperatures. These models are in Table 2. The statistical analysis involved linear and nonlinear regression. The parameters were estimated with use of SigmaPlot v12.2 (SPSS Inc., Chicago, IL, USA).

### 3.6. Comparison Criteria for Sorption Models

Four quantitative criteria were used.

a. The coefficient of determination (*R*^2^)

b. The standard error of the model (s)

(15) $$s=(\frac{\Sigma \left(yi-\hat{y}i\right)}{n-2}{)}^{0.5}$$

where yi is the measured value, $\hat{y}i$ is the predicted value from model, and n is the number of data.

c. The mean relative error (MRE)

(16) $$\mathrm{MRE}=\frac{100\%}{n}\Sigma \left|\frac{yi-\hat{y}i}{yi}\right|$$

d. Predicted errors sum of square (PRESS)

The PRESS was used to evaluate the predictive performance of sorption models [31]. The criterion was derived from the predictive error, e_-i_. When a dataset was used to compare the predictive ability of a model, the i observation was withdrawn, and the remaining n-1 data were used to estimate the parameters of the model. The i data was substituted into this regression model to calculated the predicted value $\hat{y}$_-i_. The difference between the original y_i_ value and y_-i_ value was called the predictive error, e_-i_. The sum of the square e_-i_, $\Sigma {\left(e_{\text{-i}}\right)}^{2}$ is called the PRESS. 

Residual plots were used as the criterion to evaluate the adequacy of the models. If the residual plots presented a clean pattern, the model was considered inadequate. If the residual plots exhibited a uniform distribution, the model was considered adequate for these sorption data.

## 4. Results and Discussion

### 4.1. Sorption Isotherm of C. Morifolium Flowers 

**Figure 2:**
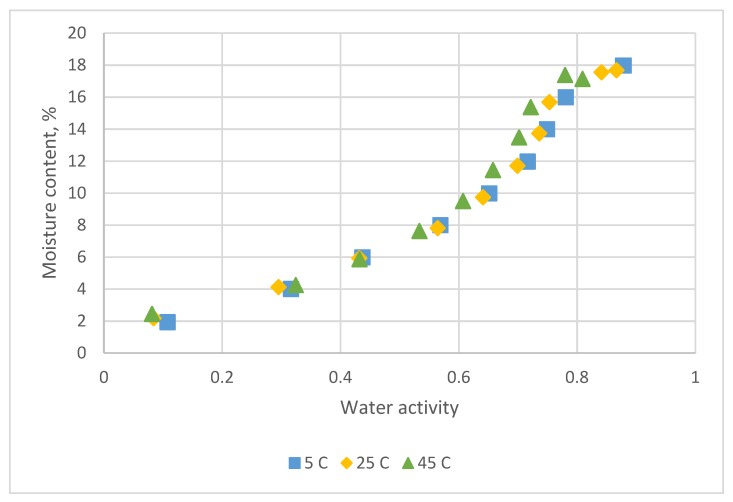
Sorption data for *C. morifolium* flowers obtained at 5 °C, 25 °C and 45 °C by the equilibrium relative humidity method.

The adsorption data for *C. morifolium* flowers at three temperatures is shown in Figure 2. 

The equilibrium time for each equilibrium relative humidity test was 12 h. The required time to reach the weight balance of the EMC method was 40–60 days for withered leaves, black and green tea [32] and 60–80 days for persimmon leaves [33]. The equilibrium relative humidity method could save the required experimental time.

The sorption isotherms of *C. morifolium* flower was a sigmoid form and reflected a type II BET classification [1,10].

The sorption temperature had a marginal effect on the adsorption isotherm (Figure 2). The reason may be explained by its rewetting history from fried samples [1].

**Figure 3:**
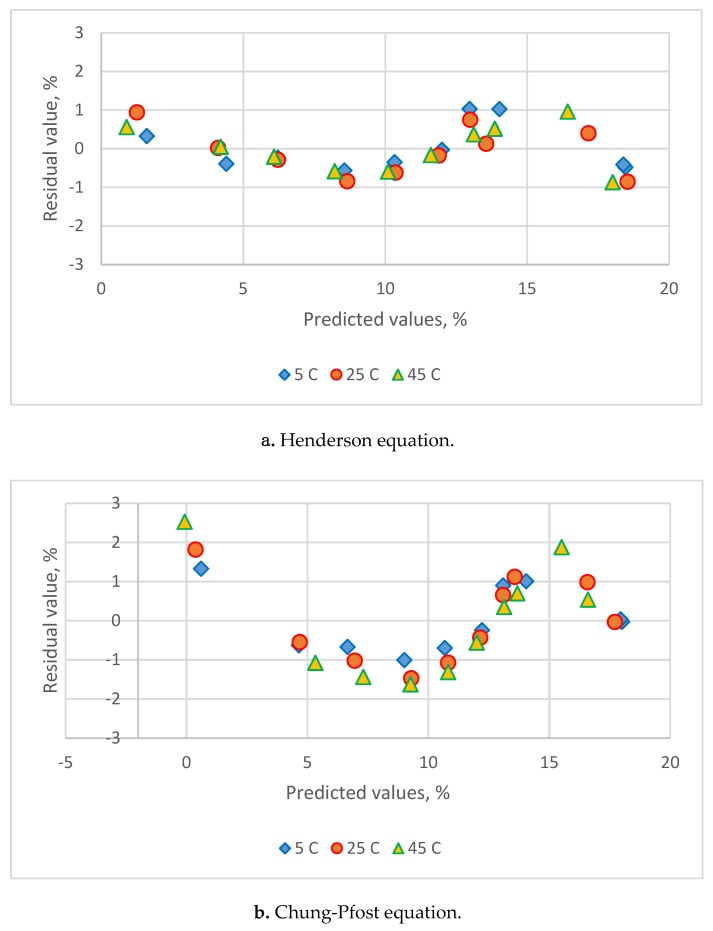

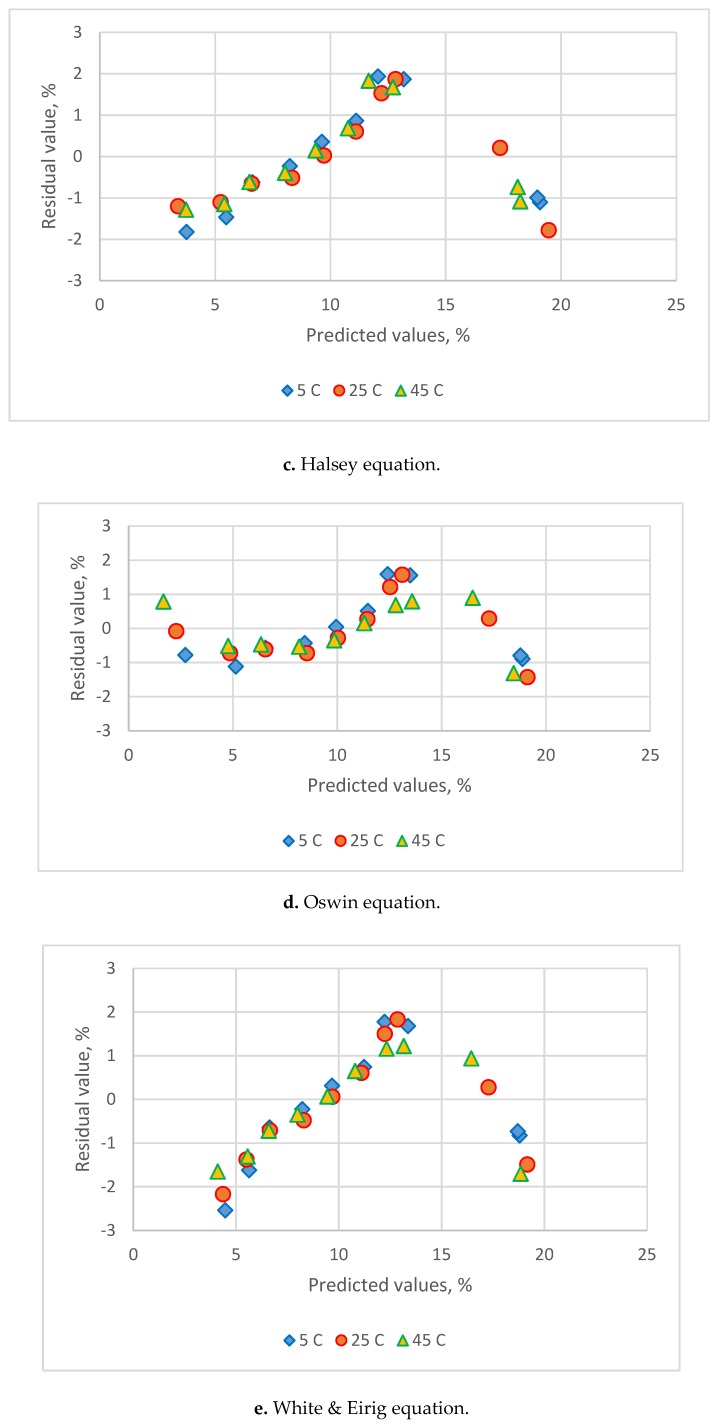

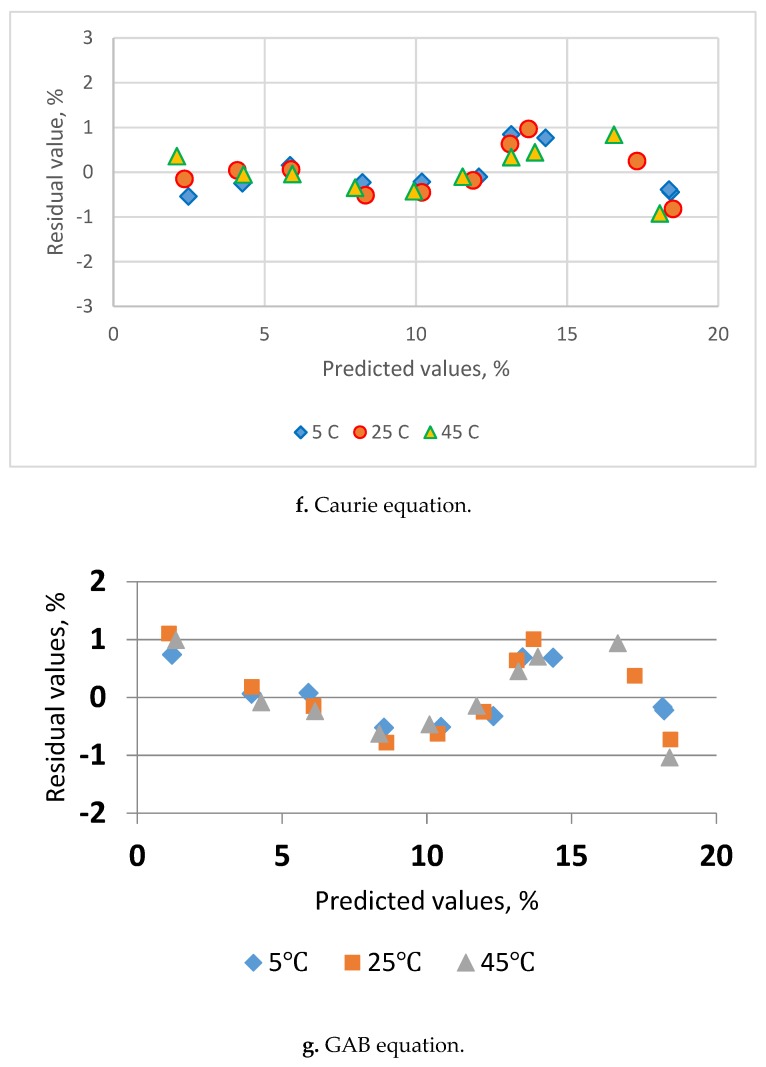
Residual plots for the sorption isotherm equations for sorption data of *C. morifolium* flowers obtained at three temperatures. (**a**) Henderson eq., (**b**) Chung-Pfost eq., (**c**) Halsey eq., (**d**) Oswin eq., (**e**) White & Eirig eq., (**f**) Caurie eq., (**g**) GAB eq.

Table 3 lists the estimated parameters and comparison statistics for seven models. The residual plots are shown in Figure 3. The Caurie equation had higher value for R^2^ and lower value for s, MRE and PRESS. The residual plots at five temperatures all showed a uniform distribution with the Henderson and Caurie equations. The Chung-Pfost, Halsey, Oswin, White & Eirig and GAB equations gave lower R^2^ and higher value for s, MRE and PRESS. The residual plots all showed a systematic pattern. These five equations could not be served as adequate equations for adsorption data of *C. morifolium* flowers.

### 4.2. Sorption Isotherm of A. Formosanus HAYATA

**Figure 4:**
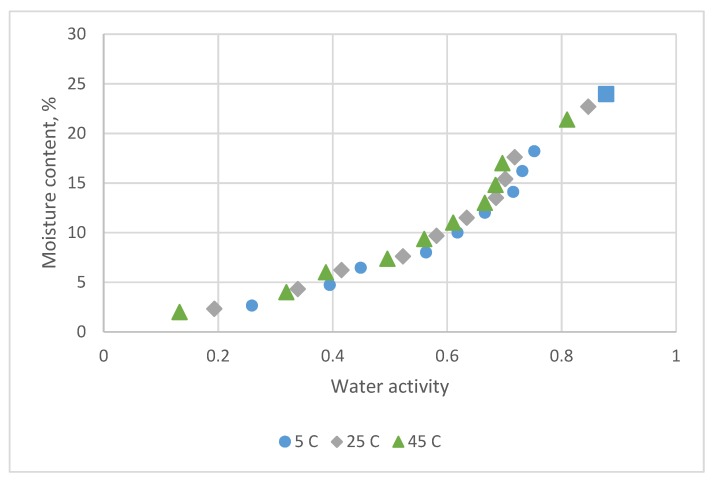
Sorption data for *A. formosanus* Hayata obtained at 5 °C, 25 °C and 45 °C by the equilibrium relative humidity method.

Figure 4 displays the adsorption EMC data for *A. formosanus* Hayata at three temperatures. The sorption isotherms of this product have a sigmoid form and display the type II on BET classification [1,10].

The sorption temperature only had a little effect on the adsorption data. Table 4 indicates the estimated parameters and comparison statistics for seven models. The results are similar to those of *C. morifolium* flowers. The Chung-Pfost, Halsey, Oswin, White & Eirig and GAB equations had lower R^2^ and higher value for s, MRE and PRESS. The residual plots all presented a systematic pattern. 

The Henderson and Caurie equations all conferred a uniform distribution of residual plots. However, the quantitative criteria were not consistent. For example, for the adsorption data at 5 °C, 15 °C and 25 °C, the Caurie equation had larger values of R^2^ and smaller value of s and PRESS. The Henderson equation, on the other hand, had a smaller value for MRE. Both equations could describe well the adsorption data for *A. formosanus* Hayata.

### 4.3. Predictive Ability of Component Model for Two Herbs

**Figure 5:**
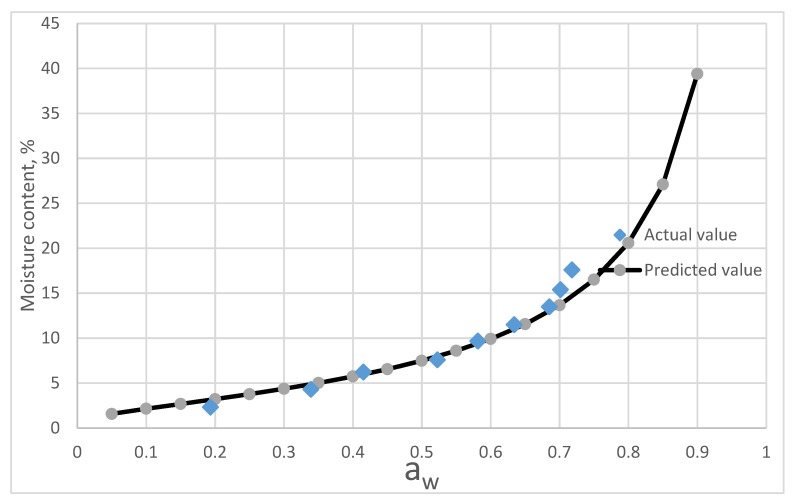
Comparing of the predictive values from the component model and the actual isotherm moisture of *A. formosanus* Hayata.

**Figure 6:**
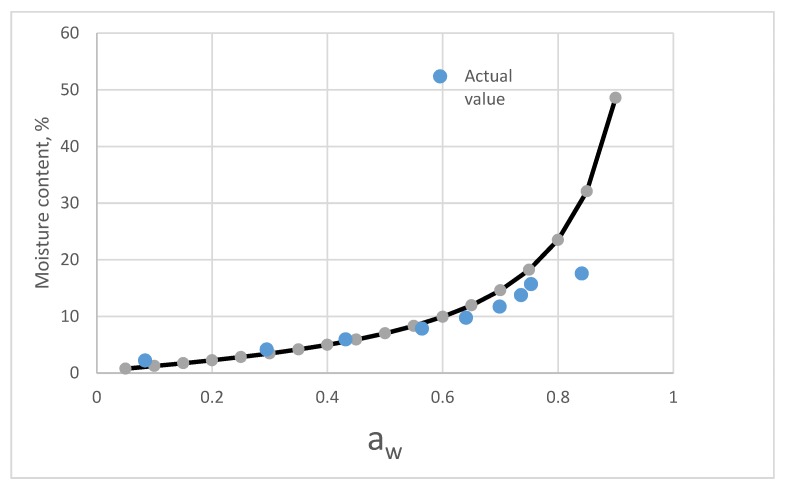
Comparing of the predictive values from the component model and the actual isotherm moisture of C. *Morifolium* flower.

The chemical composition of C. *morifolium* flower and *A. formosanus* Hayata is listed in Table 5. The prediction curves calculated from equation (8–13) and the fitting curves of actual measurement values are shown in Figure 5 and Figure 6.

Figure 5 shows that component model exhibits a good agreement with the sorption isotherms of *A. formusanus* Hayata below 0.7 a_w_. Above 0.7 a_w_, the predicted values by component model was lower than that of measurement values.

A comparison of predictive values of component model and actual sorption isotherm of C. *morifolium* flower is shown in Figure 6. Below 0.65 a_w_, both values were close. However, the predictive values of component model increase rapidly due to the high moisture value of the vacuolar component. 

### 4.4. Predictive Ability of Component Model for Other Products

The chemical composition of five agricultural products is listed in Table 6. These ratios of chemical compositions were taken from the literature, as was the sorption isotherm of these products.

**Figure 7:**
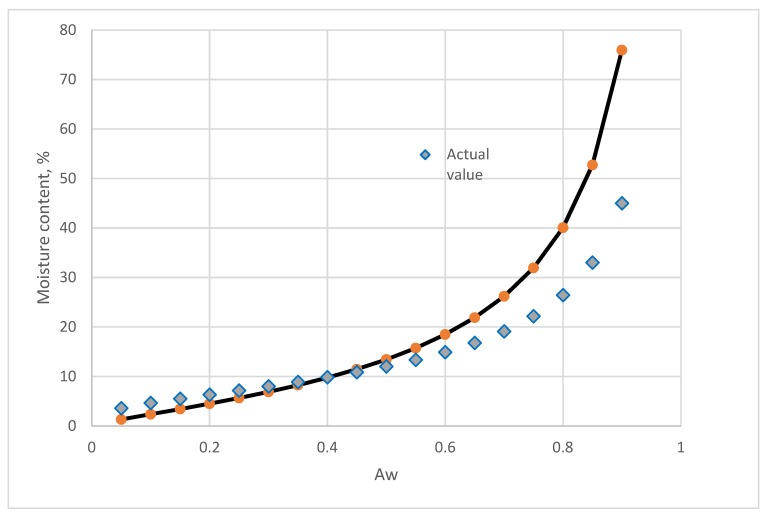
Comparing of the predictive values from the component model and the actual isotherm moisture of raw bamboo.

The predictive values and actual isotherm of raw bamboo are shown in Figure 7. Below 60 % RH, the predictive value and sorption isotherm is closed. As the RH increases, the discrepancy of the moisture content between the predictive values increased.

**Figure 8:**
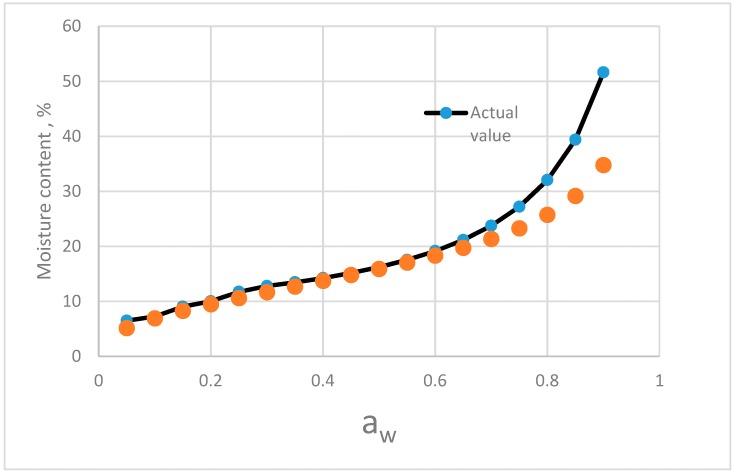
Comparing of the predictive values from the component model and the actual isotherm moisture of elecampe (*Inula helenium* L.).

The comparison results of the predictive values and actual isotherm of elecampe (*Inula helenium* L.) [34] are given in Figure 8. Below 0.65 a_w_, the predictive value and actual values of sorption isotherm are closed. In the higher a_w_ range, the discrepancy of the moisture content between predictive values increased. 

**Figure 9:**
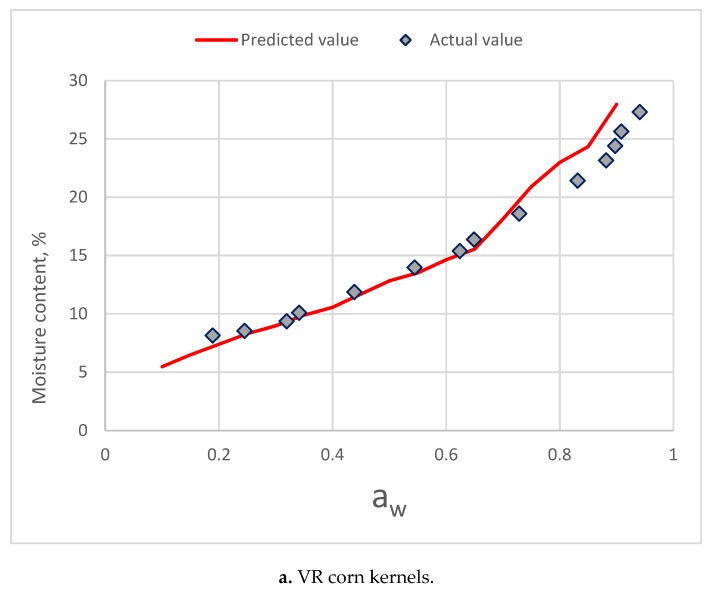

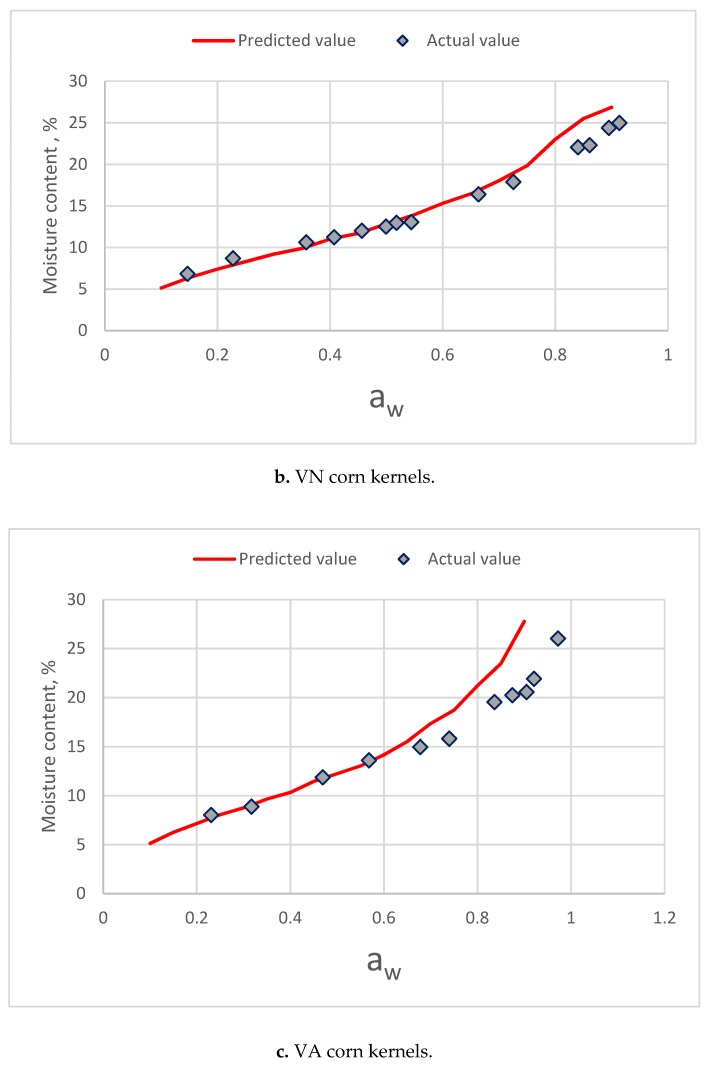
Comparing of the predictive values from the component model and the actual isotherm moisture of three varieties of corn kernel. (a. VR corn kernels; b. VN corn kernels; c. VA corn kernels).

The results of comparison for three corn varieties is presented in Figure 9. Below 0.7 a_w_, predictive values of component model were close to the sorption isotherms [34]. The moisture of predictive value increased rapidly for three corn varieties.

The results of the comparison between predictive values of component model and sorption isotherm of two herbs and five products were similar. As a_w_ higher than 0.7, the component model showed a good predictive ability. When a_w_ was higher than 0.7, the predictive moisture content increased rapidly with an increase of a_w_.

Crapiste and Rotstein [9] proposed a starchy-component model to predict isotherms from components. They suggested that their model could be applied over the entire range of moisture content. However, the results of this study indicated that this component model was valid in the a_w_ range below 0.7. The failure is the higher a_w_ range of this component model might be due to the interaction of these components is the higher a_w_ range. 

From the viewpoint of practical application, the a_w_ range below 0.7 is the main range for the processing of agricultural products and food stuffs. Pathogenic microorganisms cannot develop at a_w_ smaller than 0.6. With a_w_ at 0.3, the products is in stable with respect to non-enzymatic browning, lipid oxidation, enzyme activity and other microbial parameters [10], so the good predictive ability of component model at the a_w_ range smaller than 0.7 could be helpful for food engineering and for the food industry.

## 5. Conclusions

A component model was proposed to predict the moisture sorption isotherm data. The moisture sorption isotherm of *C. morifolium* flowers and *A. formosanus* Hayata was determined using an equilibrium relative humidity method at five temperatures. Seven sorption isotherm models were selected to evaluate the fitting ability and prediction performance for these products. Sorption temperature did not have a significant effect on the adsorption isotherms for the three samples. The Caurie and Henderson equations could be used for *C. morifolium* flowers. Considering the quantitative criteria, the Caurie equation is the best. The Henderson and Caurie equations were adequate for sorption isotherms of *A. formosanus* Hayata, but the quantitative criteria were not consistent. The isotherm data of raw bamboo, elecampe and three varieties of corn kernels from the literature were adopted to validate the component model. The component model showed a good predictive ability within an a_w_ range smaller than 0.7. Considering the practical application, the a_w_ range below 0.7 is the main range for the processing of agricultural products, so the predictive values of this component model could be helpful for food engineering and for the food industry.

## Figures and Tables

**Table 1:** Specifications of the Shinyei THT-V2-112-73-A2 transmitter.

Specification	Temperature Sensor	RH Meter
Sensing element	RTD Pt 100 Ohm	Micro-molecule HP-MQ
Operating range	0–50 °C	10–100 % RH
Accuracy before calibrating	±0.5 °C	±2 % RH
Precision	0.1 °C	0.1 % RH
Accuracy after calibrating	±0.15 °C	±0.7 % RH

RH: relative humidity.

**Table 2:** Moisture sorption isotherms models fitted to the sorption data.

Model	Equations	References
Henderson	$M=a_{0}{\left(-\ln(1-\mathrm{aw}\right)}^{a_{1}}$	Henderson [26]
Chung-Pfost	$M=b_{0}+b_{1}\ln\left(-\ln\mathrm{aw}\right)$	Chung and Pfost [20]
Halsey	$M=c_{0}{\left(\frac{-1}{\ln\mathrm{aw}}\right)}^{c_{1}}$	Halsey [27]
Oswin	$M=d_{0}{\left(\frac{aw}{1-\mathrm{aw}}\right)}^{d_{1}}$	Oswin [28]
White & Eirig	$M=\frac{1}{e_{0}+e_{1}*\mathrm{aw}}$	Castillo et al. [29]
Caurie	$M=f_{0}Exp\left(f_{1}*\mathrm{aw}\right)$	Castillo et al. [29]
GAB	$M=\frac{A*B*C*\mathrm{aw}}{\left(1-b*\mathrm{aw}\right)\left(1-B*\mathrm{aw}+B*C*\mathrm{aw}\right)}$	Van der Berg, [30]

Where a_0_, …, f_0_, a_1_, …, f_1_, A, B, and C are parameters of the equation, M is equilibrium moisture content (%, dry basis), and a_w_ is the water activity in decimal.

**Table 3:** Estimated parameters and evaluating criteria of six models used for adsorption data at five temperatures for *C. morifolium* flowers.

Temp.		5 °C	15 °C	25 °C	35 °C	45 °C
Henderson	a_0_	9.8934	10.1609	10.1559	10.4075	10.8141
	a_1_	0.8358	0.9196	0.8595	0.9956	1.0105
	R^2^	0.9386	0.9788	0.9821	0.9806	0.9789
	s	0.6393	0.8606	0.7764	0.7938	0.8120
	MRE	6.0383	8.5932	8.7059	10.2933	10.0554
	PRESS	4.9916	12.1870	8.1442	8.2361	8.9443
	Residual plot	Uniform	Uniform	Uniform	Uniform	Uniform
Chung-Pfost	b_0_	5.5080	5.7531	5.8960	5.9260	6.1290
	b_1_	−6.1120	−6.4110	−6.0820	−6.6020	−6.7451
	R^2^	0.9790	0.9531	0.9661	0.9361	0.9250
	s	0.8660	1.2861	1.1630	1.4422	1.5306
	MRE	13.0605	17.5566	16.5122	19.6941	21.4310
	PRESS	11.7280	28.5011	23.4440	41.1900	52.1751
	Residualplot	Pattern	Pattern	Pattern	Pattern	Pattern
Halsey	c_0_	5.9427	5.7082	5.9209	5.4476	5.5654
	c_1_	1.5704	0.6639	0.6126	0.7576	0.7842
	R^2^	0.9438	0.9536	0.9526	0.9764	0.9737
	s	1.4243	1.2710	1.2632	0.8753	0.9080
	MRE	19.2776	13.2668	13.9556	7.6251	7.5905
	PRESS	25.6329	42.4362	27.9405	16.7586	17.7869
	Residual plot	Pattern	Pattern	Pattern	Pattern	Pattern
Oswin	d_0_	7.3971	7.3641	7.5094	7.2905	7.5193
	d_1_	0.4731	0.5396	0.4994	0.6045	0.6214
	R^2^	0.9687	0.9702	0.9715	0.9826	0.9794
	s	1.0622	1.0188	0.9788	0.7514	0.8034
	MRE	11.8940	6.7995	7.5281	7.0538	8.7580
	PRESS	14.4665	24.8822	16.3209	10.6988	11.9955
	Residual plot	Pattern	Pattern	Pattern	Pattern	Pattern
White & Eirig	e_0_	0.2472	0.2560	0.2483	0.2698	0.2648
	e_1_	−0.2208	−0.2389	−0.2263	−0.2632	−0.2116
	R^2^	0.9399	0.9397	0.9420	0.9576	0.9516
	s	1.4737	1.4493	1.3964	1.1739	1.2312
	MRE	22.6821	19.4736	18.9198	14.9920	15.3631
	PRESS	24.9283	63.0580	31.8313	31.8209	34.7616
	Residual plot	Pattern	Pattern	Pattern	Pattern	Pattern
Caurie	f_0_	1.8739	1.6968	1.8784	1.5901	1.6464
	f_1_	2.6012	2.7997	2.6398	2.9493	2.9602
	R^2^	0.9926	0.9862	0.9904	0.9908	0.9908
	s	0.5195	0.6927	0.5697	0.5455	0.5370
	MRE	8.8299	4.6566	3.8838	4.8482	4.2159
	PRESS	3.3214	8.4673	4.8398	4.7978	5.1792
	Residual plot	Uniform	Uniform	Uniform	Uniform	Uniform
GAB	A	6.1289	2.8515	2.8434	1.4477	3.1852
	B	1.3142	2.0917	2.1447	3.3973	1.9537
	C	0.6442	0.7811	0.7457	0.8807	0.8257
	R^2^	0.9923	0.9805	0.9835	0.9833	0.9803
	s	0.5634	0.8815	0.7959	0.786	0.839
	MRE	6.7162	8.7490	9.3550	8.2197	9.4683
	PRESS	3.7444	28.4023	10.4076	16.6284	16.3538
	Residual plot	Pattern	Pattern	Pattern	Pattern	Pattern

MRE, mean relative error; PRESS, predicted errors sum of square.

**Table 4:** Estimated parameters and evaluating criteria of six models used for adsorption data at five temperatures for *A. formosanus* Hayata.

Temp.		5 °C	15 °C	25 °C	35 °C	45 °C
Henderson	a_0_	11.1085	11.2013	11.7819	11.9449	12.1878
	a_1_	1.0682	1.1939	1.0687	1.1718	1.1162
	R^2^	0.9771	0.9915	0.9841	0.9930	0.9876
	s	1.0785	0.6406	0.8610	0.5601	0.7261
	MRE	4.1659	4.3288	5.0736	6.4703	7.3331
	PRESS	36.7479	8.3019	19.5555	4.7611	10.3204
	Residual plot	Uniform	Uniform	Uniform	Uniform	Uniform
Chung-Pfost	b_0_	4.1801	4.1701	5.1377	5.422	5.813
	b_1_	−9.5630	−10.0561	−9.328	−9.368	−8.993
	R^2^	0.9766	0.9711	0.9710	0.9540	0.9501
	s	1.1071	1.1751	1.1800	1.4240	1.4502
	MRE	10.3642	12.8435	13.0605	17.7778	19.8735
	PRESS	17.5531	22.1391	21.831	37.8920	40.4921
	Residual plot	Pattern	Pattern	Pattern	Pattern	Pattern
Halsey	c_0_	6.3600	5.6542	6.2743	5.7114	5.9771
	c_1_	0.6749	0.8224	0.7433	0.8781	0.8446
	R^2^	0.9200	0.9647	0.9411	0.9779	0.9638
	s	2.0171	1.3065	1.6567	0.9920	1.2398
	MRE	20.8838	14.0305	18.1594	10.9186	13.6242
	PRESS	189.6994	54.1043	11.6067	28.4231	54.7304
	Residual plot	Pattern	Pattern	Pattern	Pattern	Pattern
Oswin	d_0_	7.9799	7.5396	8.1977	7.8989	8.1917
	d_1_	0.5792	0.6871	0.6177	0.7013	0.6802
	R^2^	0.9045	0.9795	0.9634	0.9890	0.9791
	s	2.0171	0.9951	1.3052	0.6993	0.9417
	MRE	15.8019	9.1047	12.1321	5.3923	7.0749
	PRESS	119.8241	29.7879	63.4608	13.2440	28.1725
	Residual plot	Pattern	Pattern	Pattern	Pattern	Pattern
White & Eirig	e_0_	0.2248	0.2476	0.2266	0.2492	0.2394
	e_1_	−0.2099	−0.2434	−0.2172	−0.2522	−0.2396
	R^2^	0.9161	0.9435	0.9220	0.9423	0.9304
	s	2.0653	1.6539	1.9283	1.5002	1.7178
	MRE	24.0033	20.7259	24.6922	20.7785	24.2669
	PRESS	268.41	106.2321	206.2235	77.7422	149.9925
	Residual plot	Pattern	Pattern	Pattern	Pattern	Pattern
Caurie	f_0_	1.3805	1.1989	1.5561	1.3618	1.5218
	f_1_	3.2662	3.5179	3.1850	3.4184	3.2744
	R^2^	0.9864	0.9938	0.9884	0.9956	0.9905
	s	0.8298	0.5459	0.7366	0.4442	0.6335
	MRE	6.5503	4.7103	6.3682	4.6476	5.6618
	PRESS	18.0125	5.1170	14.0032	3.2240	8.8902
	Residual plot	Uniform	Uniform	Uniform	Uniform	Uniform
GAB	A	156146.6	9696.467	11806.88	7.9452	65.9138
	B	0.007007	0.0269	0.0269	1.0877	0.3634
	C	0.5401	0.5952	0.5232	0.8255	0.6303
	R^2^	0.9875	0.9933	0.9895	0.9941	0.9894
	s	0.8451	0.6023	0.7433	0.5455	0.7122
	MRE	4.7524	4.6051	5.3836	5.3507	6.9848
	PRESS	132.6791	14.4521	65.4748	12.0731	34.3074
	Residual plot	Pattern	Pattern	Pattern	Pattern	Pattern

**Table 5:** Chemical composition of C. *morifolium* flower and *A. formosamus* Hayata.

Component	*C. Morifolium* Flower (16)	*A. formosamus* Hayata (17)
Sugar	0.4923	0.098
Ash	0.077	0.02
Fiber	0.13384	0.23
Starch	0.07692	0.0
Protein	0.1892	0.07
Oil	0.0311	0.01

Note: The unit of components is decimal.

**Table 6:** Chemical composition of several agricultural products.

Component	Raw Bamboo [14]	Elecampe [34]	Corm V_R_ [35]	Corm V_n_ [35]	Corm V_A_ [35]
Sugar	0.1964	0.0	0.03965	0.01508	0.02674
Ash	0.1265	0.053	0.01925	0.01607	0.015394
Fiber	0.1429	0.01	0.03579	0.03946	0.036445
Starch	0.4688	0.874	0.75185	0.77221	0.73766
Protein	0.0045	0.0775	0.11147	0.10573	0.12969
Oil	0.0	0.0	0.04199	0.05144	0.05408

Note: The unit of components is decimal.
